# Tolerance and accumulation of lithium in *Apocynum pictum* Schrenk

**DOI:** 10.7717/peerj.5559

**Published:** 2018-08-29

**Authors:** Li Jiang, Lei Wang, Lei Zhang, Changyan Tian

**Affiliations:** 1 Key Laboratory of Biogeography and Bioresource in Arid Land, Xinjiang Institute of Ecology and Geography, Chinese Academy of Sciences, Urumqi, Xinjiang, China; 2 Turpan Eremophytes Botanical Garden, Chinese Academy of Sciences, Urumqi, Xinjiang, China; 3 State Key Laboratory of Desert and Oasis Ecology, Xinjiang Institute of Ecology and Geography, Chinese Academy of Sciences, Urumqi, Xinjiang, China

**Keywords:** Bioconcentration factor, *Apocynum pictum*, Lithium, Phytoremediation, Germination, Translocation factor

## Abstract

Primarily, lithium (Li) resource development and wider application of Li-ion batteries result in Li pollution and concomitantly poses increasing and inevitable problems to environmental health and safety. However, information is rare about the scope of the remediation of Li contaminated soil. *Apocynum venetum* is already proved to be a Li-accumulator with high Li tolerance and accumulation (Jiang et al., 2014). However, it is not clear whether *Apocynum pictum*, another species of the same genus with the same uses as *A. venetum*, is also a Li-accumulator. We investigated germination, growth and physiological responses of *A. pictum* to different levels of LiCl. Germination was not significantly affected by low Li concentration (0–100 mmol L^−1^). As LiCl increased from 100 to 400 mmol L^−1^, both germination percentage and index decreased gradually. For germination of *A. pictum* seeds, the critical value (when germination percentage is 50%) in LiCl solution was 235 mmol L^−1^, and the limit value (when germination percentage is 0%) was 406 mmol L^−1^. *A. pictum* could accumulate >1,800 mg kg^−1^ Li in leaves, and still survived under 400 mg kg^-1^ Li supply. The high Li tolerance of *A. pictum* during germination and growth stage was also reflected by activity of α-amylase and contents of soluble sugar, proline and photosynthetic pigments under different Li treatments. The bioconcentration factors (BCF) (except control) and translocation factors (TF) were higher than 1.0. High tolerance and accumulation of Li indicated that* A. pictum* is Li-accumulator. Therefore, this species could be useful for revegetation and phytoremediation of Li contaminated soil.

## Introduction

Lithium (Li), the lightest alkali metal, is normally present in trace amounts in the environment (Shahzad et al., 2017). As the beneficial and detrimental effects to living organisms depending on the dose of Li, more attention should be paid to the increase of Li contents in soil caused by industrial activities, use and disposal of Li-containing products (Schrauzer, 2002). The increase of toxic hazard is mainly caused by the dispose of used-up Li-ion batteries (Aral & Vecchio-Sadus, 2008; Robinson et al., 2018). Li is listed as a pollutant that causes environmental harm in irrigation water supplies (Australian Capital Territory Parliamentary Counsel, 2005).

In the consideration of Li toxicity and its impact on health and environment, several remediation methods have been proposed to reduce its toxicity and remediate Li contaminated soil. These approaches include: (1) identification and use of Li tolerant genotypes, accumulator and hyperaccumulator; (2) organic matter addition to soils; (3) application of chelators or chaperones; (4) *Ex-situ* and *in-situ* immobilization techniques; (5) soil stabilization and solidification (Shahzad et al., 2016). Among these methods, phytoremediation is inexpensive and can reduce secondary pollution compared with traditional physical and chemical approaches (Cluis, 2004; Franzaring et al., 2016). However, lack of necessary information about Li accumulator and hyperaccumulator is the limitation factor of this technology. Screening some Li-accumulating plant species is the first step to remediate Li-contaminated soil using phytoremediation.

Although Li is a non-essential metal for plant growth, the growth-stimulating effects of Li at low concentration are reported. For example, fresh biomass was increased by 15% in maize treated with 5 mg L^−1^ Li (Hawrylak-Nowak, Kalinowska & Szymańska, 2012). Meanwhile, Li induces considerable reduction in the growth of plants as the Li-salts are highly toxic (Franzaring et al., 2016). Citrus plants are sensitive to Li (Aral & Vecchio-Sadus, 2008). However, plants in the Asteraceae and Solanaceae families have high tolerance to Li (Schrauzer, 2002; Kabata-Pendias & Mukherjee, 2007). Thus, Li influences plant growth in both stimulation and reduction ways, depending on Li concentration and plant species.

*Apocynum pictum*, perennial halophytic herb, mainly distributes in salt-barren zone, desert margins, and riversides of the northwestern region of China (Flora of China Editorial Committee, 1995). Like *Apocynum venetum*, another species of the same genus, *A. pictum* is also a popular daily beverage and commonly used as traditional Chinese medicine to calm the liver, sooth nerves, and promote dieresis (Thevs et al., 2012). Modern pharmacological studies have demonstrated that many pharmacological effects of both plants are similar to those of Li (Editorial Committee of Chinese Pharmacopoeia, 2010; Khasraw et al., 2012). *A. venetum* is already proved to be a Li-accumulator (Jiang et al., 2014), so we hypothesized that *A. pictum* is also a Li-accumulator.

The aim of this study was to examine the accumulation capacity of Li in *A. pictum* and also to examine Li toxicity tolerance ability. Specifically, three questions were addressed: (1) What is Li tolerance for *A. pictum* at germination stage? (2) Does an increase in soil Li contents inhibit the growth of *A. pictum* and reduce photosynthetic pigments? (3) Are Li contents in different organs of *A. pictum* affected by elevated soil Li?

## Materials and Methods

### Germination experiment

Mature fruits were collected from plants of *A. pictum* cultivated at Fukang Field Research Station of the Chinese Academy of Sciences (44°17′N, 87°56′E; 460 m a.s.l.), located in the southern part of the Junggar Basin of Xinjiang Province, China, in early October 2016. Fruits were allowed to dry naturally for two weeks. Then, the seeds were detached from fruits and stored dry at room temperature.

Germination experiment was performed on 4th November 2016. Seeds were placed on two layers of filter paper in 5-cm-diameter Petri dishes. Filter paper was moistened with 2.5 mL of 0 (distilled water), 25, 50, 100, 150, 200, 300 and 400 mmol L^−1^ LiCl (Tianjin Fuchen Chemical Reagent Co., Ltd). Four replicates of 25 seeds were used per treatment. The lids were sealed with Parafilm and incubated at 25 °C under a 14/10 h light/dark photoperiod for 15 days. Lithium solutions were changed every 5th day. The criteria for germination were the radicle length ≥1 mm. The number of germinated seeds was counted and germinated seeds were removed from the Petri dishes at each counting. The velocity of germination was estimated using a modified Timson’s index of germination velocity (Khan & Ungar, 1984).

### Physiological parameter

Seeds of *A. pictum* were incubated on two layers of filter paper in 5-cm-diameter Petri dishes. Filter paper was moistened with 0 (distilled water control), 25, and 300 mmol L^−1^ LiCl. Petri dishes were sealed with parafilm and incubated at 25 °C under a 14/10 h light/dark photoperiod. Incubation was terminated after 2, 4, 6, 8, 10 or 12 d.

To extract α-amylase, fresh seeds (0.2 g) were homogenized with 8.0 mL distilled water. Activity of α-amylase was measured spectrophotometrically at 540 nm (Li, 2000). After fresh seeds (0.2 g) were homogenized with 10 mL of 10% trichloroacetic acid, the malondialdehyde (MDA) content was determined according to the method of (Duan et al., 2005). After fresh seeds (0.2 g) were homogenized with 10.0 mL distilled water, soluble sugar content was measured spectrophotometrically at 630 nm (Li, 2000). After fresh seeds (0.2 g) were homogenized with 5.0 mL of 3% sulfonyl salicylic acid, proline content was measured spectrophotometrically at 520 nm (Li, 2000).

### Pot experiment

The pots were placed in a greenhouse at Fukang Field Research Station of the Chinese Academy of Sciences. Gray desert topsoil (0–20 cm) was collected and used as the growth substrate. The soil was air-dried and homogenized, after which it was sieved through 4-mm mesh. Soil pH was 8.47, organic matter 0.92 %, total N 0.96 g kg^−1^, available N 89.54 mg kg^−1^, available P 9.24 mg kg^−1^, available K 341.16 mg kg^−1^, and Li 25.75 mg kg^−1^. Each plastic pot (35 cm height, 33 cm diameter) was filled with 10 kg of air-dry soil mixed with different contents of Li (solution prepared by dissolving analytical grade LiCl): 0 (CK without external Li), 50, 200, and 400 mg kg^−1^. To simulate the Li impacted soils, pot soil was homogenized for four weeks with the additive Li. Two *A. pictum* rhizome cuttings of similar size (9–10 cm height, ca. 1 cm diameter) were transplanted into each pot on 14 April, 2012. There were eight replicates for each treatment, and they were arranged in a randomized block design. Each pot received 10 g granular lawn fertilizer (Osmocote 301; Scotts, Marysville, OH, USA; 15N: 11P: 13K: 2Mg) once at the beginning as the basic fertilizer. Each pot was irrigated with 1 L distilled water every two day.

After 90 days of cultivation, *A. pictum* plants were harvested and separated into roots, stems, and leaves. The leaves and stems were rinsed with distilled water three times and then blotted with filter paper. The roots were dipped in a CaCl_2_ solution for 5 minutes to get rid of trace elements adsorbed at the root surface and then rinsed three times with distilled water. Fresh material was oven-dried at 80 °C for 48 h. Afterwards the samples were weighed, ground, and sieved through 60-mesh.

Photosynthetic pigments were extracted in darkness at 4 °C with 5 ml mixture of acetone and ethanol (v/v = 1:1) for 48 h. After filtering, chlorophyll a (Chl a), chlorophyll b (Chl b) and carotenoid (Car) contents were determined by spectrophotometry using 663, 645, and 470 wavelength. Pigment concentrations were calculated following Lichtenthaler (1987).

0.2 g plant sample was digested with 10 ml concentrated HNO_3_ (50%) for 12 h in triangular flask. Then, 3 ml H_2_O_2_ (30%) was added and kept in a temperature control electric cooker (Lab Tech EH35A) at 150 °C for 10 min. After cooled to 20 °C, 5 ml HNO_3_ (1%) and 1ml H_2_O_2_ (30%) were added. Sample was digested at 150 °C until the white smoke emitted fully and the solution was transparent. After cooled to 20 °C, the solution was diluted to 50 ml with HNO_3_ (1%). Li content was estimated with an inductively coupled plasma spectroscopy (Perkin Elmer SciexDRC II) in the range of 670–783 nm (Kalinowska, Hawrylaknowak & Szymańska, 2013), and determined at 670.783 nm.

Bioconcentration factors (BCF) is defined as the ratio of the metal concentration in the root to that in the soil; Translocation factors (TF) is defined as the ratio of the metal concentration in the shoot to that in the root (Baker & Brooks, 1989).

### Statistical analysis

Germination percentage data were arcsine transformed before statistical analysis. One-way ANOVA was used to test the effect of different Li concentrations on germination, dry weight, pigment contents, Li concentrations, BCF, and TF. Duncan’s test was used to determine differences among treatments when ANOVA showed significant effects (*P* < 0.05).

## Results

### Germination characteristics of *A. pictum* seeds

**Figure 1:**
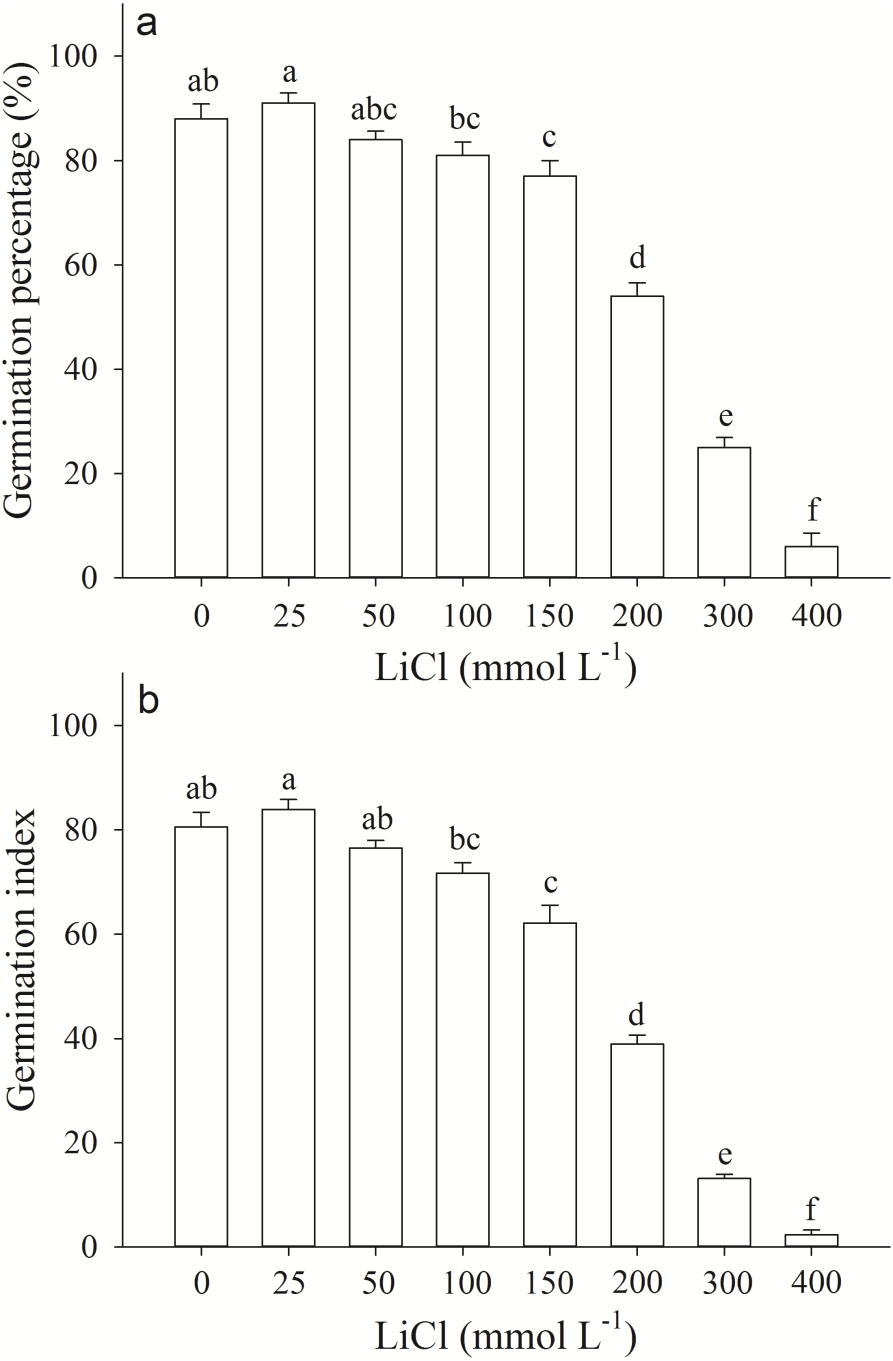
Germination response of *Apocynum pictum* seeds to LiCl. Effects of LiCl solution on germination percentage (A) and germination index (B) of *Apocynum pictum* seeds. Different letters indicate significant differences in germination percentages or germination index among different LiCl concentrations with Duncan’s test (*P* < 0.05).

Germination percentage and index of *A. pictum* seeds were significantly affected by LiCl. With the increase of concentration of LiCl, germination percentages and indexes decreased (Fig. 1). Germination percentages of *A. pictum* seeds were reduced from 91% to 6% under 0–400 mmol L^−1^ LiCl (Fig. 1A). Under low to moderate concentration of LiCl (25–100 mmol L^−1^), *A. pictum* seeds achieved high germination percentage (>80%). Even under 200 mmol L^−1^ LiCl, germination percentage could reach 54%. The regression equation of the relationship between germination percentages of *A. pictum* seeds and LiCl concentrations is: *y* =  − 0.0002777*x*^2^ − 0.1141*x* + 92.03 (*R*^2^ = 0.9538). The simulated critical value (when germination percentage is 50%) is 235 mmol L^−1^. The simulated limit value (when germination percentage is 0%) is 406 mmol L^−1^.

**Figure 2:**
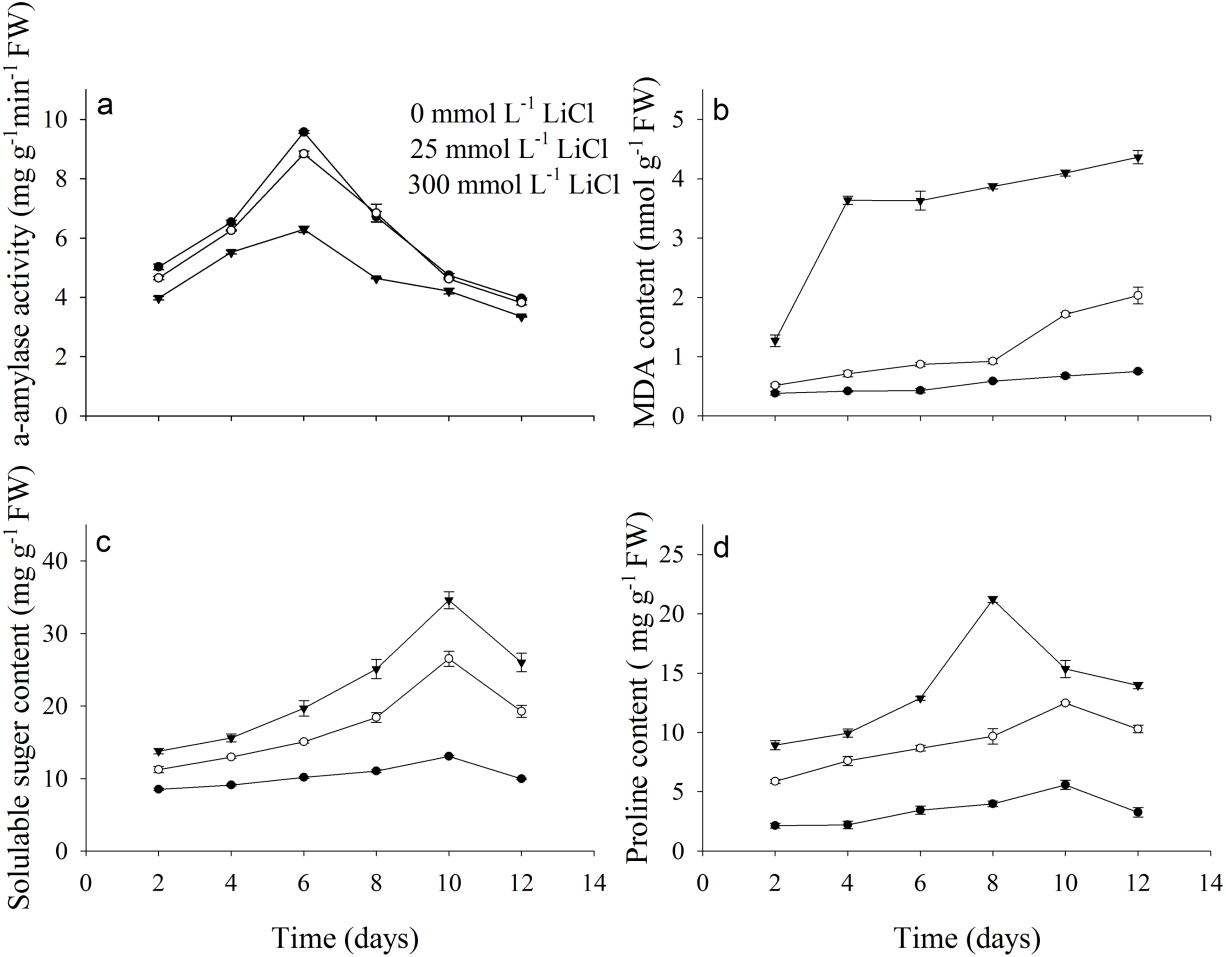
Physiological parameters of *Apocynum pictum* to LiCl during germination. Effects of LiCl concentration on (A) *α*-amylase activity, (B) MDA, (C) soluble sugar and (D) proline contents in seeds of *Apocynum pictum* during germination.

Activity of α-amylase, contents of MDA, soluble sugar and proline were dramatically affected by LiCl (Fig. 2). Least α-amylase activity was noted under 300 mmol L^−1^ LiCl (Fig. 2A). Contents of MDA, soluble sugar and proline under 300 mmol L^−1^ LiCl were the highest (Figs. 2B–2D). Activity of α-amylase fist increased and then decreased under Li treatment and was highest after 6 days of incubation. Contents of MDA increased with treated time in LiCl.

### Growth and pigment contents of *A. pictum* plants

**Figure 3:**
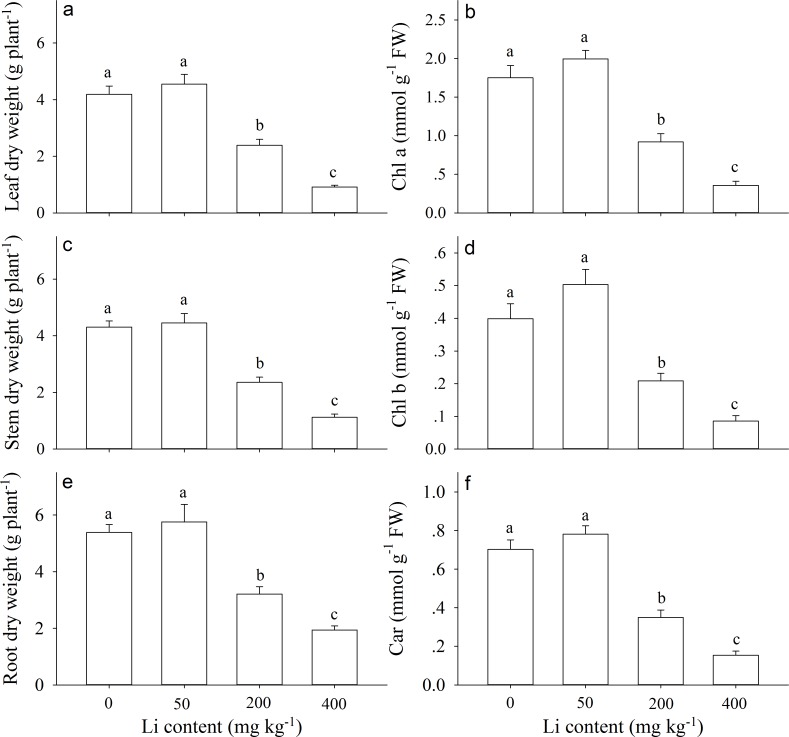
Growth and physiological responses of *Apocynum pictum* to additional soil Li. Effects of additional soil Li on leaf dry weight (A), stem dry weight (C), root dry weight (E), chlorophyll a content (B), chlorophyll b content (D), and car content (F) of *Apocynum pictum*. Different letters indicate significant differences in each measures among different additional soil Li contents with Duncan’s test (*P* < 0.05).

Dry weight of leaf, stem and root of *A. pictum* did not significantly change at 50 mg kg^−1^ additional soil Li, but only significantly decreased at 200 and 400 mg kg^−1^ additional Li (Figs. 3A, 3C, 3E). When compared with 0 mg kg^−1^ Li, the reductions of leaves dry weight were 42.90% and 78.15% under 200 and 400 mg kg^−1^ Li treatment, respectively; the reductions of stems dry weight were 45.34% and 74.02% under 200 and 400 mg kg^−1^ Li compared with control respectively; the reductions of roots dry weight were 40.41% and 64.02% under 200 and 400 mg kg^−1^ Li treatment respectively.

Pigment contents (*Chl a*, *Chl b*, and *Car*) were not significantly affected by 50 mg kg^−1^ additional Li (Figs. 3B, 3D & 3F). However, for plants grown at 200 and 400 mg kg^−1^ additional Li, there were significant reductions in contents of *Chl a, Chl b*, and *C* ar compared with control. When compared with 0 mg kg^−1^ Li, the reductions of *Chl a* contents were 29.96% and 78.30% under 200 and 400 mg kg^−1^ Li treatment, respectively; the reductions of *Chl b* contents were 24.09% and 76.75% under 200 and 400 mg kg^−1^ Li respectively; the reductions of *Car* contents were 32.49% and 76.75% under 200 and 400 mg kg^−1^ Li respectively.

### Li accumulation and translocation of *A. pictum* plants

**Figure 4:**
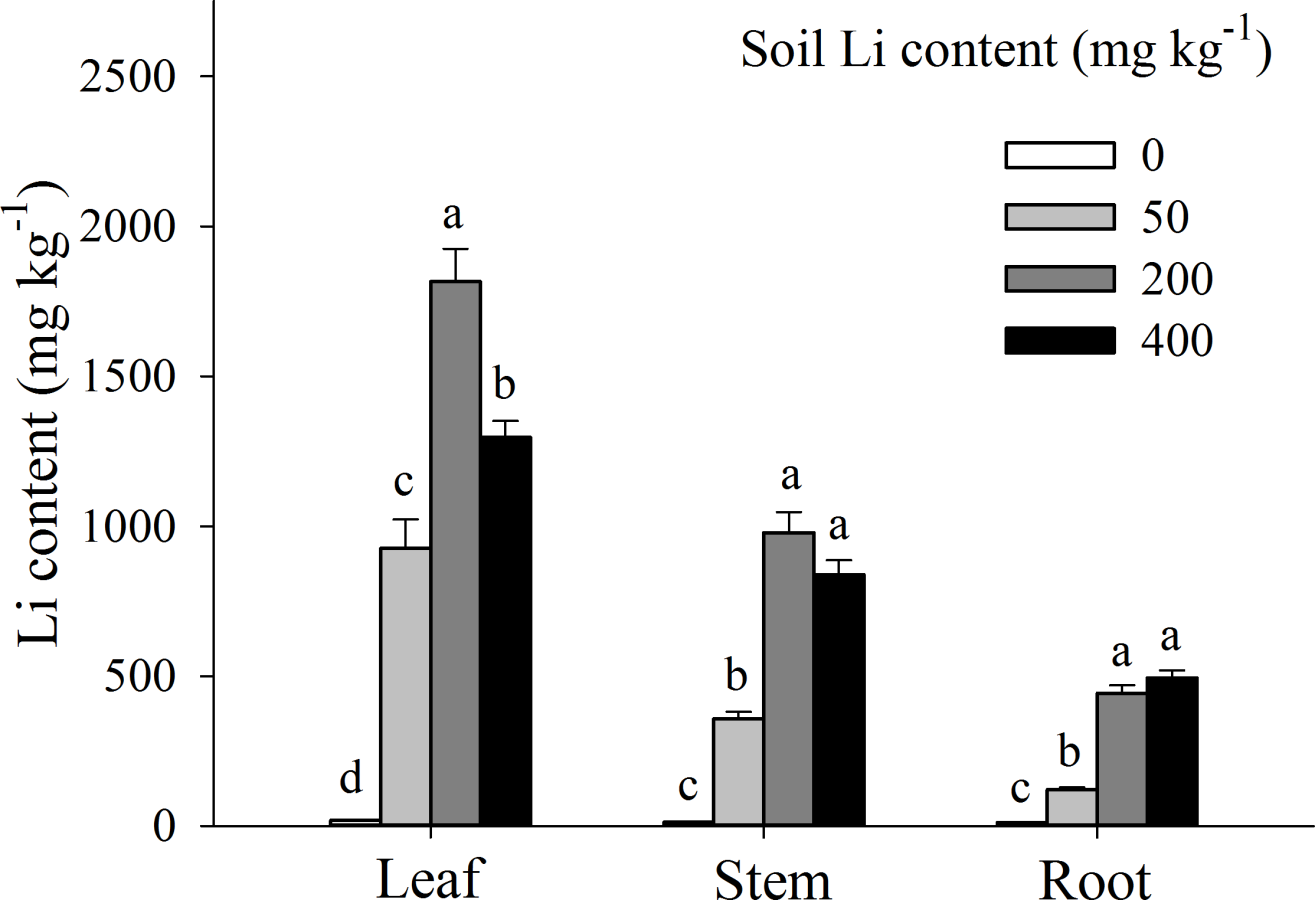
Li contents in different organs of *Apocynum pictum* in responses to additional soil Li. Effects of additional soil Li on Li contents in leaf, stem and root of *Apocynum pictum*. Different letters indicate significant differences in Li contents of the same plant organ with Duncan’s test (*P* < 0.05).

Li accumulation in different plant tissues were significantly influenced by external Li concentration in soil, however among different tissues, Li concentration in leaves was significantly higher than that in stems and roots (*P* < 0.05; Fig. 4). Moreover, in leaves and stem tissues, Li concentration was increased, marked from 0 to 200 mg kg^−1^ additional Li (*P* < 0.05), and then deceased from 200 to 400 mg kg^−1^ additional Li. Contrarily, in *A. pictum* roots, Li concentration was proportionally increased with an increase in additional Li in soil (0 to 400 mg kg^−1^) (Fig. 4). When compared with Li contents in leaves, the decrements of Li contents in roots were 39.62%, 86.80%, 75.60%, and 61.82% under 0, 50, 200 and 400 mg kg^−1^ Li treatment, respectively.

**Figure 5:**
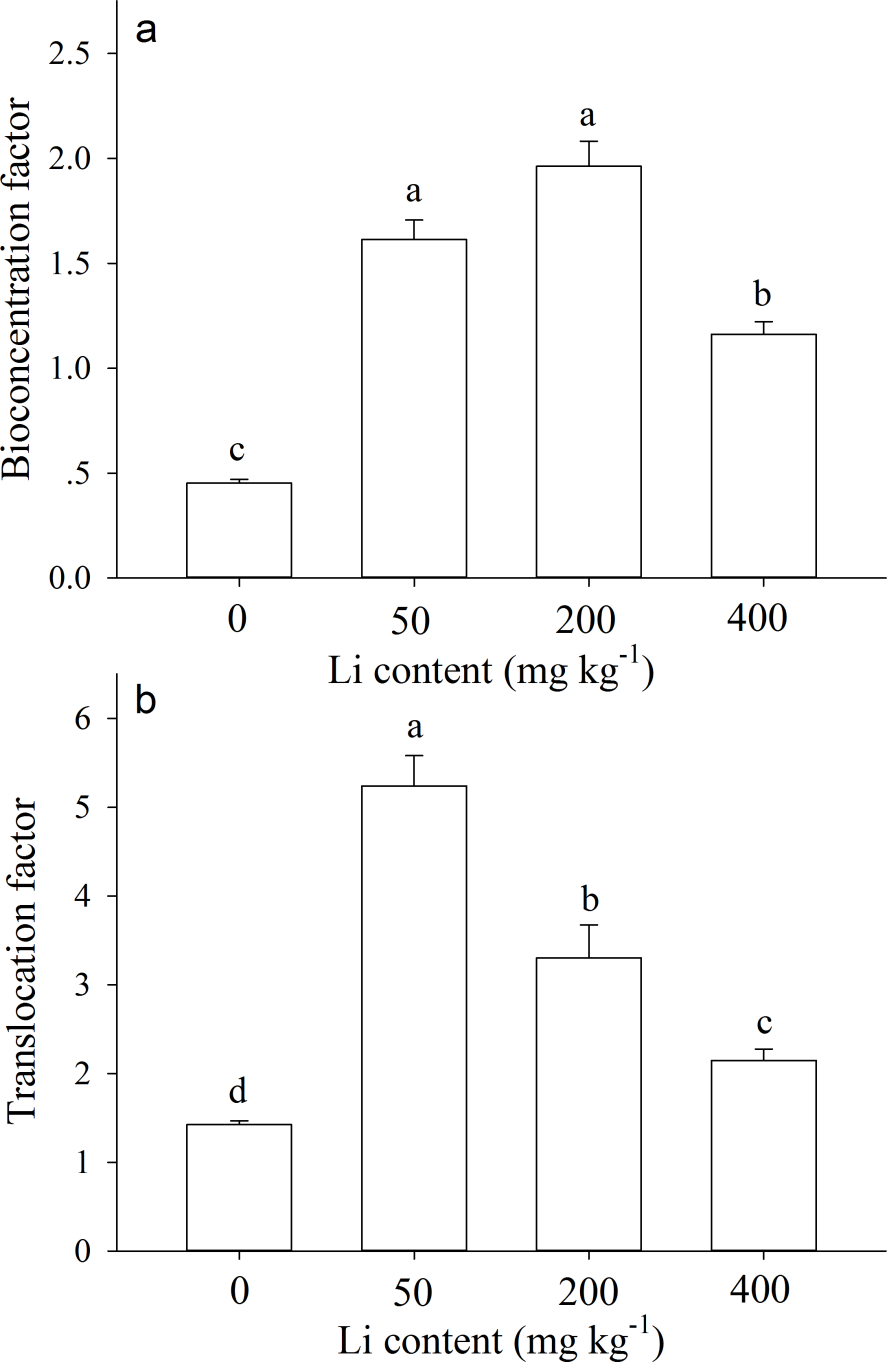
Bioconcentration factor and translocation factor of *Apocynum pictum* to additional soil Li. Effects of additional soil Li on bioconcentration factor (BCF) (A) and translocation factor (TF) (B) of *Apocynum pictum*. Different letters indicate significant differences in BCF or TF among different additional soil Li contents with Duncan’s test (*P* < 0.05).

The bioconcentration factors (BCF) and translocation factors (TF) values were greater than 1.0 for most Li treatments (except control). After 90 d of cultivation, BCF value of *A. pictum* at 0 (control), 50, 200, and 400 mg kg^−1^ additional Li was 0.45, 1.61, 1.96, 1.16, respectively (Fig. 5A). TF value at 0, 50, 200, and 400 mg kg^−1^ additional Li was 1.42, 5.24, 3.30, 2.15, respectively (Fig. 5B).

## Discussion

Although germination, plant growth, and ecophysiological responses of *A. pictum* to environmental factors have been studied extensively (Ma et al., 2003; Thevs et al., 2012; Shi et al., 2014), these data are the first in which tolerance and accumulation characteristics of Li in *A. pictum* plants have been documented. In addition, our data indicate that seeds of *A. pictum* have high Li tolerance during germination. Our results of plant growth and Li accumulation traits indicated *A. pictum* as a potential Li-accumulator.

Cultivation of Li-accumulator in Li polluted soil might be an economical and eco-friendly method for revegetation (Shahzad et al., 2016). The information of germination responses of Li-accumulator seeds to different Li concentrations is vital for soil Li phytoremediation purpose. For Li-sensitive plants, such as *Oryza sativa* and *Cicer arietinum*, germination percentages were affected by 0.72 mmol L^−1^ (Gupta, 1974). Even for potential Li accumulator *Brassica carinata*, germination percentage was <50% at 120 mmol L^−1^ LiCl (Li et al., 2009). For Li-accumulator *Apocynum venetum*, the critical value (when germination percentage is 50%) in LiCl solution is 196 mmol L^−1^ (Jiang, Wang & Tian, 2018). Our results showed germination percentage of *A. pictum* was 54% at 200 mmol L^−1^ LiCl, and showed that *A. pictum* exhibited high Li tolerance during germination stage (Fig. 1).

The high Li tolerance of *A. pictum* during germination stage was also reflected by different physiological parameters. α-amylase plays an important role in the starch metabolism during germination (Muralikrishna & Nirmala, 2005). Activity of α-amylase is an indicator of catalytic efficiency of hydrolysis of starch into sugars (Salieri, Vinci & Antonelli, 1995). There was no significantly difference in the activity of α-amylase under 0 and 25 mmol L^−1^ LiCl. This result indicated the 25 mmol L^−1^ LiCl did not dramatically affect the changes in α-amylase involved in starch breakdown during germination compared with 0 mmol L^−1^ Li treatment. Organic solutes play an important role in osmotic adjustment in plants under salt stress (Martino et al., 2003). *A. pictum* seeds during germination under different Li treatments accumulated high concentrations of soluble sugar and proline (Fig. 2). This clearly highlighted that *A. pictum* can tolerate high Li concentration by increasing the accumulation contents of organic solutes. *A. pictum* accumulated proline and soluble sugar as cheap osmoticum to ameliorate Li induced toxicity. Further studies are required to investigate molecular basis of Li tolerance mechanisms in *A. pictum.*

Plants of *A. pictum* showed high tolerance to Li stress because plant growth and pigment contents were not significantly affected by 75 mg kg^−1^ soil Li. For Li tolerant species *Chloris gayana* and *Zea mays*, their growth were reduced up to 25% at 65 and 70 mg kg^−1^ soil Li, respectively (Bingham, Bradford & Page, 1964). Among different plant tissues, *A. pictum* roots accumulated small amount of Li, however, stems and leaves accumulated large amount of Li (Fig. 4). When additional soil Li reached 200 mg kg^−1^, Li content in stems and leaves of *A. pictum* was more than 950 and 1,800 mg kg^−1^, respectively (Fig. 4). Reported Li-accumulators, such as *Cirsium arvense* and *Solanum dulcamera* could accumulate 1,000 mg kg^−1^ Li in leaves (Tölgyesi, 1983). Compared with these plants, the capacity of *A. pictum* plants to accumulate Li is even better. Even compared with *A. venetum*, a newly found Li-accumulator of the same genus (Jiang et al., 2014), the capacity of *A. pictum* plants to tolerate and accumulate Li is equal. BCF and TF are key indexes used to evaluate metal accumulation efficiency in plants. BCF of *A. pictum* were 1.16–1.96 for 50–400 mg kg^−1^ additional soil Li, and TF of *A. pictum* were 2.15–5.24 for 50–400 mg kg^−1^ additional soil Li, respectively, which were higher than 1.0 (the critical level for accumulator).

## Conclusions

Current evidence shows that *A. pictum* seeds have high Li tolerance and *A. pictum* plants have high Li tolerance and accumulation. Growth and physiological results confirm that *A. pictum* is a tolerant species to Li. Contents of Li in roots, stems and leaves, BCF and TF indicate that this species has the basic traits of a Li-accumulator. High Li tolerance of *A. pictum* seeds and plants implies that sowing this species directly is possible in Li contaminated soil and may be useful for phytoremediation and revegetation of Li contaminated sites. However, it is not clear whether other environmental factors such as soil physicochemical properties have a positive or negative effect in determining Li resistance and absorbing. Future research on multiple factor experiments will help to answer this question.

##  Supplemental Information

10.7717/peerj.5559/supp-1 Supplemental Information 1 Germination data of *Apocynum pictum* to different LiCl concentrations Four replicates of 25 seeds germinated under 0 (distilled water), 25, 50, 100, 150, 200, 300 and 400 mmol L ^−1^ LiCl. The number of germinated seeds was counted every day. Each data point indicates the number of germinated seeds.

10.7717/peerj.5559/supp-2 Supplemental Information 2 Physiological parameters of *Apocynum pictum* to different LiCl concentrations during germination Four replicates of 25 seeds germinated under 0 (distilled water), 100 and 300 mmol L ^−1^ LiCl. The physiological parameters of germinated seeds was tested every two days. Each data point indicates the physiological index of germinated seeds.

10.7717/peerj.5559/supp-3 Supplemental Information 3 Biomass, contents of photosynthetic pigments and Li accumulation in different organs of *Apocynum pictum* Each data point indicates weight, contents of photosynthetic pigments or Li in different organs of *Apocynum pictum*. Eight replicates for each treatment.

## Additional Information and Declarations

### Data Availability

The following information was supplied regarding data availability: The raw data are provided in the Supplemental Files.

## References

[ref-1] Aral H, Vecchio-Sadus A (2008). Toxicity of lithium to humans and the environment—a literature review. Ecotoxicology & Environmental Safety.

[ref-2] Australian Capital Territory Parliamentary Counsel (2005). Environment Protection Regulation 2005. http://www.legislation.act.gov.au/sl/2005-38/20051118-21565/pdf/2005-38.pdf.

[ref-3] Baker AJ, Brooks RR (1989). Terrestrial higher plants which hyperaccumulate metallic elements—-a review of their distribution, ecology and phytochemistry. Biorecovery.

[ref-4] Bingham FT, Bradford GE, Page AL (1964). Toxicity of lithium. California Agriculture.

[ref-5] Cluis C (2004). Junk-greedy greens: phytoremediation as a new option for soil decontamination. Bioteach Journal.

[ref-6] Duan BL, Lu YW, Yin CY, Junttila Q, Li CY (2005). Physiological responses to drought and shade in two contrasting *Picea asperata* populations. Physiologia Plantarum.

[ref-7] Editorial Committee of Chinese Pharmacopoeia (2010). Chinese Pharmacopoeia, 2010 edition.

[ref-8] Flora of China Editorial Committee (1995). Flora of China.

[ref-9] Franzaring J, Schlosser S, Damsohn W, Fangmeier A (2016). Regional differences in plant levels and investigations on the phytotoxicity of lithium. Environmental Pollution.

[ref-10] Gupta IC (1974). Lithium tolerance of wheat, barley, rice and gram at germination and seedling stage. Indian Journal of Agricultural Research.

[ref-11] Hawrylak-Nowak B, Kalinowska M, Szymańska M (2012). A study on selected physiological parameters of plants grown under lithium supplementation. Biological Trace Element Research.

[ref-12] Jiang L, Wang L, Mu SY, Tian CY (2014). *Apocynum venetum*: a newly found lithium accumulator. Flora.

[ref-13] Jiang L, Wang L, Tian CY (2018). High lithium tolerance of *Apocynum venetum* seeds during germination. Environmental Science & Pollution Research.

[ref-14] Kabata-Pendias HA, Mukherjee AB (2007). Trace elements from soil to human.

[ref-15] Kalinowska M, Hawrylaknowak B, Szymańska M (2013). The influence of two lithium forms on the growth, l-Ascorbic acid content and lithium accumulation in lettuce plants. Biological Trace Element Research.

[ref-16] Khan MA, Ungar IA (1984). Seed polymorphism and germination responses to salinity stress in *Atriplex triangularis* willd. Botanical Gazette.

[ref-17] Khasraw M, Ashley D, Wheeler G, Berk M (2012). Using lithium as a neuroprotective agent in patients with cancer. BMC Medicine.

[ref-18] Li HS (2000). Experimental principles and techniques of plant physiology and biochemis.

[ref-19] Li X, Gao P, Gjetvaj B, Westcott N, Gruber MY (2009). Analysis of the metabolome and transcriptome of *Brassica carinata* seedlings after lithium chloride exposure. Plant Science.

[ref-20] Lichtenthaler HK (1987). Chlorophylls and carotenoids: pigments of photosynthetic biomembranes. Methods in Enzymology.

[ref-21] Ma M, Cheng LH, An SQ, Li B (2003). Seasonal, spatial, and interspecific variation in quercetin in *Apocynum venetum* and *Poacynum hendersonii*, Chinese traditional herbal teas. Journal of Agriculture and Food Chemistry.

[ref-22] Martino CD, Delfine S, Pizzuto R, Loreto F, Fuggi A (2003). Free amino acids and glycine betaine in leaf osmoregulation of spinach responding to increasing salt stress. New Phytologist.

[ref-23] Muralikrishna G, Nirmala M (2005). Cereal alpha-amylases-an overview. Carbohydrate Polymers.

[ref-24] Robinson BH, Yalamanchali R, Reiser R, Dickinson NM (2018). Lithium as an emerging environmental contaminant: mobility in the soil-plant system. Chemosphere.

[ref-25] Salieri G, Vinci G, Antonelli ML (1995). Microcalorimetric study of the enzymatic hydrolysis of starch: an α-amylase catalyzed reaction. Analytica Chimica Acta.

[ref-26] Schrauzer GN (2002). Lithium: occurrence, dietary intakes, nutritional essentiality. Journal of the American College of Nutrition.

[ref-27] Shahzad B, Mughal MN, Tanveer M, Gupta D, Abbas G (2017). Is lithium biologically an important or toxic element to living organisms? An overview. Environmental Science & Pollution Research.

[ref-28] Shahzad B, Tanveer M, Hassan W, Shah AN, Anjum SA, Cheema SA, Ali I (2016). Lithium toxicity in plants: reasons, mechanisms and remediation possibilities—a review. Plant Physiology & Biochemistry.

[ref-29] Shi QM, Deng FY, Wu MY, Chen DD, Yin CH (2014). Study on salt tolerance of *Apocynum venetum* Linn. and *Poacynum hendersonii* (Hook.f.) Woodson at stages of seed germination and seedlings growth. North Hortic.

[ref-30] Thevs N, Zerbe S, Kyosev Y, Rozi A, Tang B, Abdusalih N, Novitskij Z (2012). *Apocynum venetum* L. and *Apocynum pictum* Schrenk (Apocynaceae) as multi-functional and multi-service plant species in Central Asia: a review on biology, ecology, andutilization. Journal of Applied Botany & Food Quality.

[ref-31] Tölgyesi G, Anke M, Baumann W, Braunlich H (1983). Distribution of lithium in Hungarian soils and plants.

